# Comparative Evaluation of Frictional Resistance Between Different Types of Ceramic Brackets and Stainless Steel Brackets With Teflon-Coated Stainless Steel and Stainless Steel Archwires: An In-Vitro Study

**DOI:** 10.7759/cureus.24161

**Published:** 2022-04-15

**Authors:** K Ranjan R Bhat, Nausheer Ahmed, Rithika Joseph, Abrar Younus A

**Affiliations:** 1 Department of Orthodontics and Dentofacial Orthopaedics, Government Dental College and Research Institute Bangalore, Bengaluru, IND

**Keywords:** teflon-coated stainless steel, ceramic bracket with metal slot, esthetic brackets, universal testing machine, scanning electron microscope, ceramic bracket, surface roughness, stainless steel, friction

## Abstract

Background

Orthodontic tooth movement relies on sliding mechanics usually achieved by sliding the archwire through brackets. Sliding causes friction which is a force resisting the relative motion of two contacting objects. Frictional resistance is undesirable in orthodontic tooth movement because the archwire might bind with the bracket and prevent tooth movement. In addition, friction causes bending of the archwire leading to unwanted tooth movement or space loss through anchorage interference, prolonging the treatment time and root resorption. This study was performed to compare the frictional resistance produced by different types of ceramic brackets and stainless steel brackets with Teflon-coated stainless steel and stainless steel archwires. The surface texture of the wire before and after friction test was also evaluated using a scanning electron microscope (SEM).

Methodology

A total of 48 samples were tested. In total, 12 premolar brackets each of stainless steel (Ortho technology, Carlsbad, CA, USA), monocrystalline ceramic (Ortho technology, Carlsbad, CA, USA), polycrystalline ceramic (Ortho technology, Carlsbad, CA, USA), and ceramic bracket with a metal slot (Ortho technology, Carlsbad, CA, USA) having an 0.022-inch slot were coupled with 0.019 × 0.025-inch stainless steel and Teflon-coated stainless steel wires. Each bracket-wire assembly was vertically mounted and clamped to the jaws of the universal testing machine. The wire was pulled across the bracket with a cross head speed of 10 mm per minute. The readings obtained were recorded. To evaluate the surface roughness, wires were examined using an SEM (in four magnifications 250×, 500×, 1,000×, and 5,000×) before and after testing.

Results

Under the testing conditions, the stainless steel bracket-stainless steel wire combination produced the least frictional resistance, and the polycrystalline ceramic bracket-stainless steel wire combination produced the highest frictional resistance. Ceramic brackets with a metal slot generated lesser friction than other types of ceramic brackets but more friction than stainless steel brackets. Moreover, for all bracket-archwire combinations, Teflon-coated wires generated reduced frictional resistance compared to stainless steel wires. The surface examination of Teflon-coated stainless steel wire and conventional uncoated stainless steel wire revealed that Teflon-coated wire had a smoother surface compared to uncoated stainless steel wire.

Conclusions

Within the limitations of this study, it was concluded that the stainless steel bracket produced the lowest frictional resistance and the polycrystalline ceramic bracket produced the highest frictional resistance. Ceramic brackets with a metal slot showed a coefficient of friction that was more than but comparable to that of stainless steel brackets. Monocrystalline ceramic brackets generated lesser friction compared to polycrystalline ceramic brackets. Further, Teflon coating of stainless steel archwires can reduce frictional resistance compared to conventional uncoated stainless steel archwires. The surface of Teflon-coated stainless steel wires was found to be smoother than uncoated stainless steel wires.

## Introduction

Orthodontic tooth movement occurs by the application of sliding mechanics using brackets and archwires. This generates friction in the appliance which opposes every movement the orthodontist desires, impeding optimum tooth movement [1]. In orthodontics, thorough knowledge about friction and its impact on treatment outcomes is crucial to achieving optimal biologic tissue response and sufficient tooth movement during sliding mechanics [2].

During retraction of the teeth, the archwire slides through the slots of the brackets inducing frictional forces, which, in turn, counteract forces generating tooth movement [3]. Higher force may be required to overcome frictional resistance at the bracket-wire interface. Increasing the orthodontic forces beyond the optimal level can lead to excessive pain, loss of anchorage, and root resorption [2].

Factors affecting friction in orthodontic tooth movement can be broadly classified into mechanical and biological factors. Mechanical factors include bracket material, slot size, bracket width, bracket/archwire angulation, archwire material, archwire shape, the surface roughness of the archwire, ligature material, and force of ligation. The biological factors that affect friction include saliva, plaque, and acquired pellicle [4].

The material with which the bracket-archwire assembly is fabricated is one of the predominant factors affecting orthodontic tooth movement using sliding mechanics. Therefore, the choice of the material is crucial to reduce the frictional forces, thereby achieving the best possible treatment outcomes. The traditional combination of stainless steel bracket and archwire outperforms any other bracket-archwire combination, with their frictional characteristics so satisfactory that they are used as the gold standard [5].

Over time, patients’ concerns switched from esthetic appearance after the treatment to during the treatment which led to the development of smaller and more invisible bracket and archwire assemblies [6]. The introduction of plastic brackets in the 1970s was the first step toward esthetic appliances. However, this system saw its early demise due to its many drawbacks, such as discoloration, malodor, and slot distortion, overpowering its only advantage of being esthetic [7].

In 1980, the field of orthodontics witnessed the first ceramic system, composed of monocrystalline and polycrystalline ceramic, which, unlike the plastic brackets, resisted staining and could withstand heavier forces without distortion [6].

Nevertheless, in practice, any material used will generate friction, making it impossible to develop a frictionless system. To provide the least hindrance to tooth movement it is advised to control friction rather than trying to eliminate it [5]. This study aims to compare and evaluate the resistance to friction between different types of ceramic brackets and stainless steel brackets with Teflon-coated stainless steel and stainless steel archwires.

## Materials and methods

Inclusion and exclusion criteria

The following four types of 0.022-in slot MBT prescription premolar brackets were tested: monocrystalline alumina, polycrystalline alumina, polycrystalline ceramic bracket with stainless steel slot, and stainless steel bracket. The Different types of brackets used were standard twin brackets with four tie wings for the use of elastomeric ligatures. The ceramic brackets are made up of aluminum oxide. A total of 12 brackets of each type were used (Table 1). Roth prescription brackets were excluded from this study. The following two types of archwires were tested: aesthetic stainless steel (Teflon-coated) and stainless steel. A total of 24 archwire segments of each wire were used (Table 2).

**Table 1:** Types of brackets used in this study.

Bracket type	Slot dimension	Quantity	Description
Monocrystalline alumina bracket	0.022 × 0.028 inch	12	Ortho technology (Carlsbad, CA, USA)
Polycrystalline alumina bracket	0.022 × 0.028 inch	12	Ortho technology (Carlsbad, CA, USA)
Polycrystalline ceramic bracket with stainless steel slot	0.022 × 0.028 inch	12	Ortho technology (Carlsbad, CA, USA)
Stainless steel bracket	0.022 × 0.028 inch	12	Ortho technology (Carlsbad, CA, USA)

**Table 2:** Types of wires used in this study.

Wire alloy	Dimension	Description
Esthetic stainless steel (Teflon-coated)	0.019 × 0.025 inch	Ortho technology (Carlsbad, CA, USA)
Stainless steel	0.019 × 0.025 inch	Ortho technology (Carlsbad, CA, USA)

**Figure 1:**
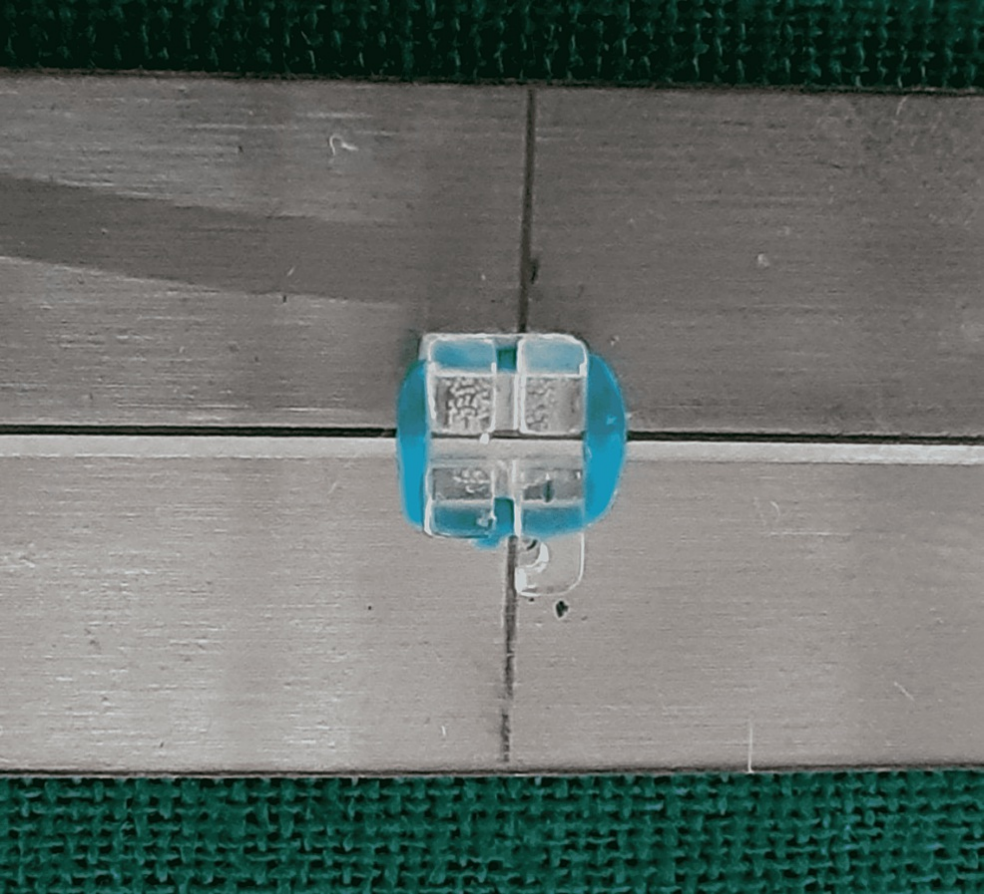
Monocrystalline ceramic bracket and stainless steel wire combination.

**Figure 2:**
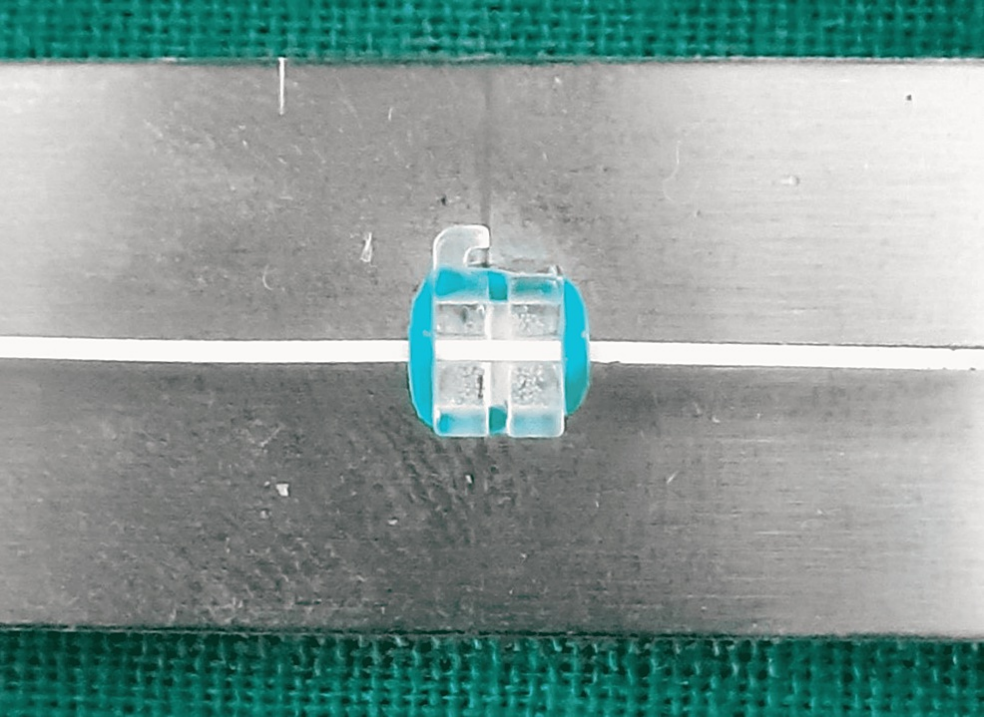
Monocrystalline ceramic bracket and Teflon-coated stainless steel wire combination.

**Figure 3:**
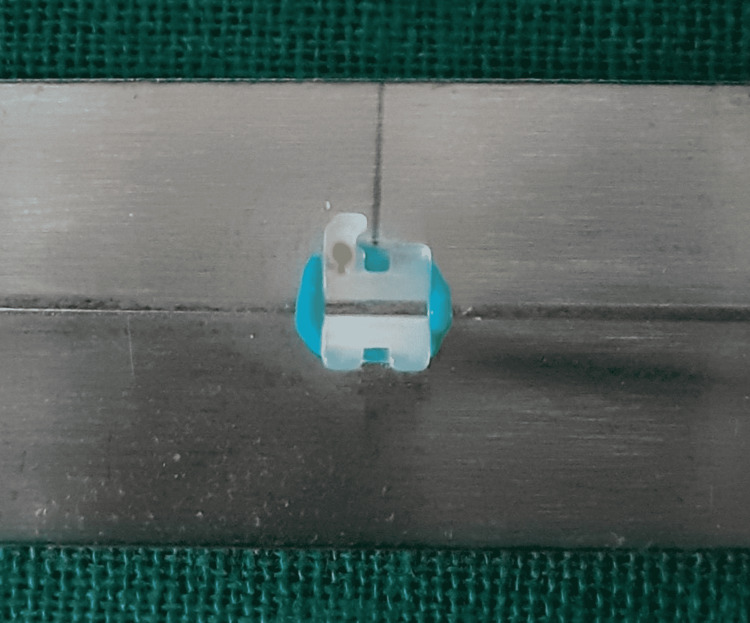
Polycrystalline ceramic bracket and stainless steel wire combination.

**Figure 4:**
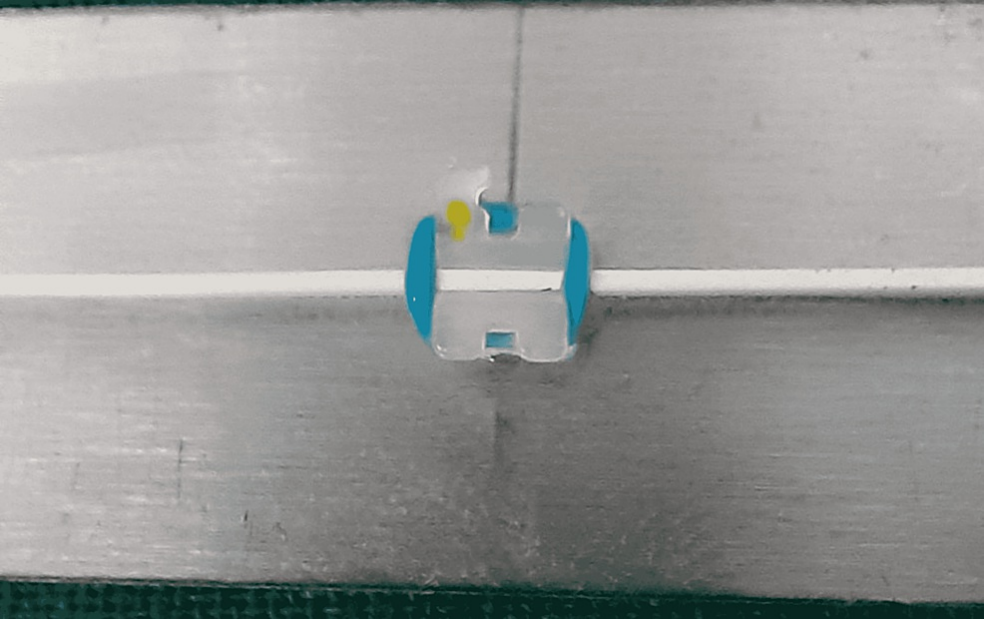
Polycrystalline ceramic bracket and Teflon-coated stainless steel wire combination.

**Figure 5:**
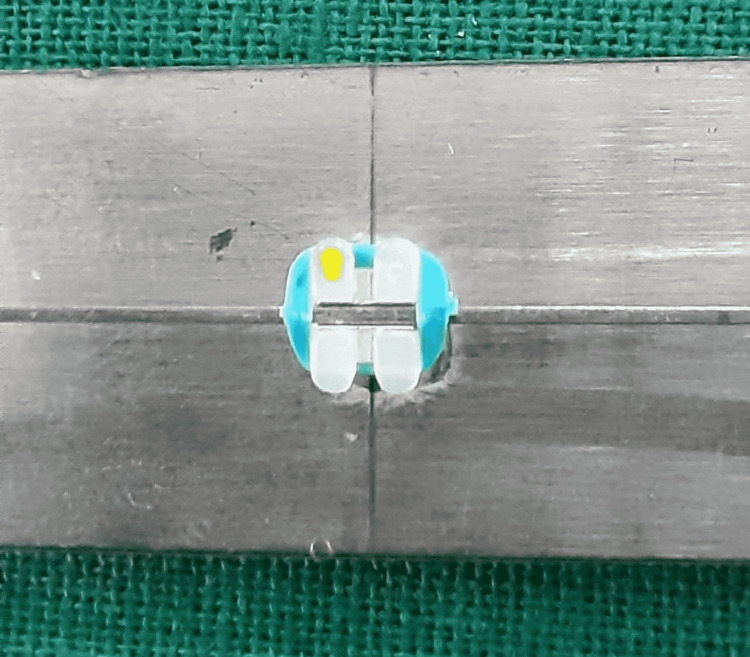
Polycrystalline ceramic bracket with a metal slot and stainless steel wire combination.

**Figure 6:**
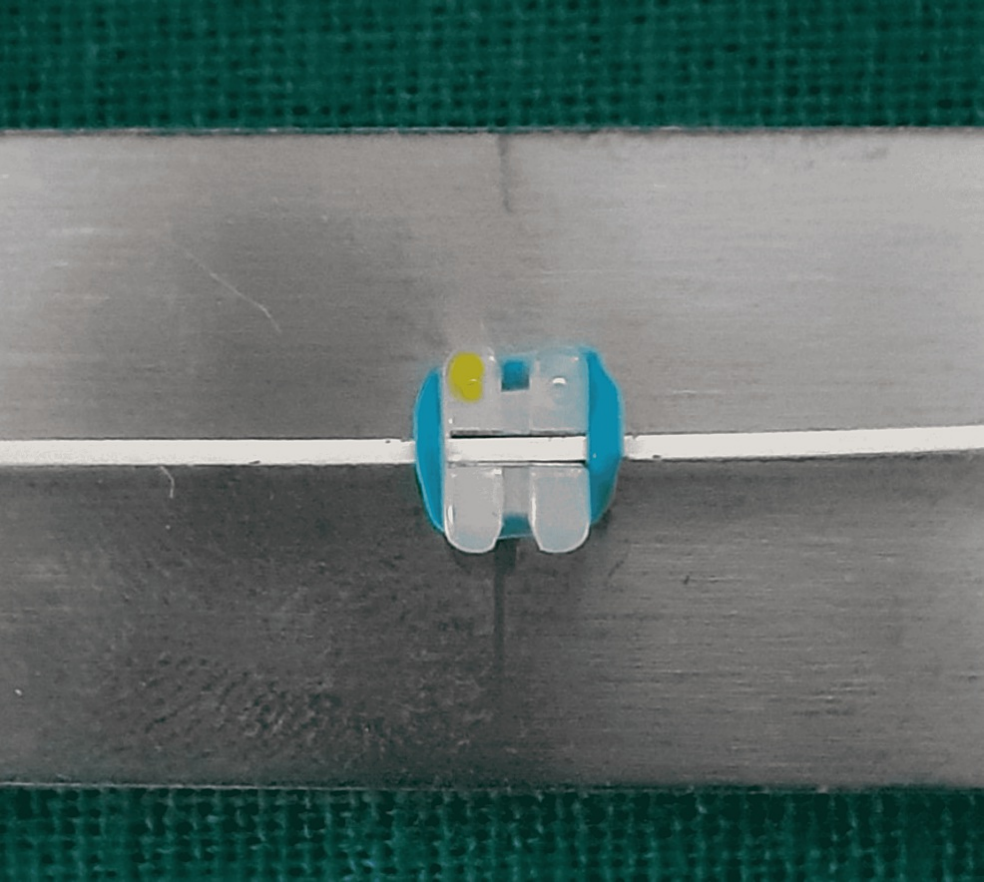
Polycrystalline ceramic bracket with a metal slot coupled with Teflon-coated stainless steel wire.

**Figure 7:**
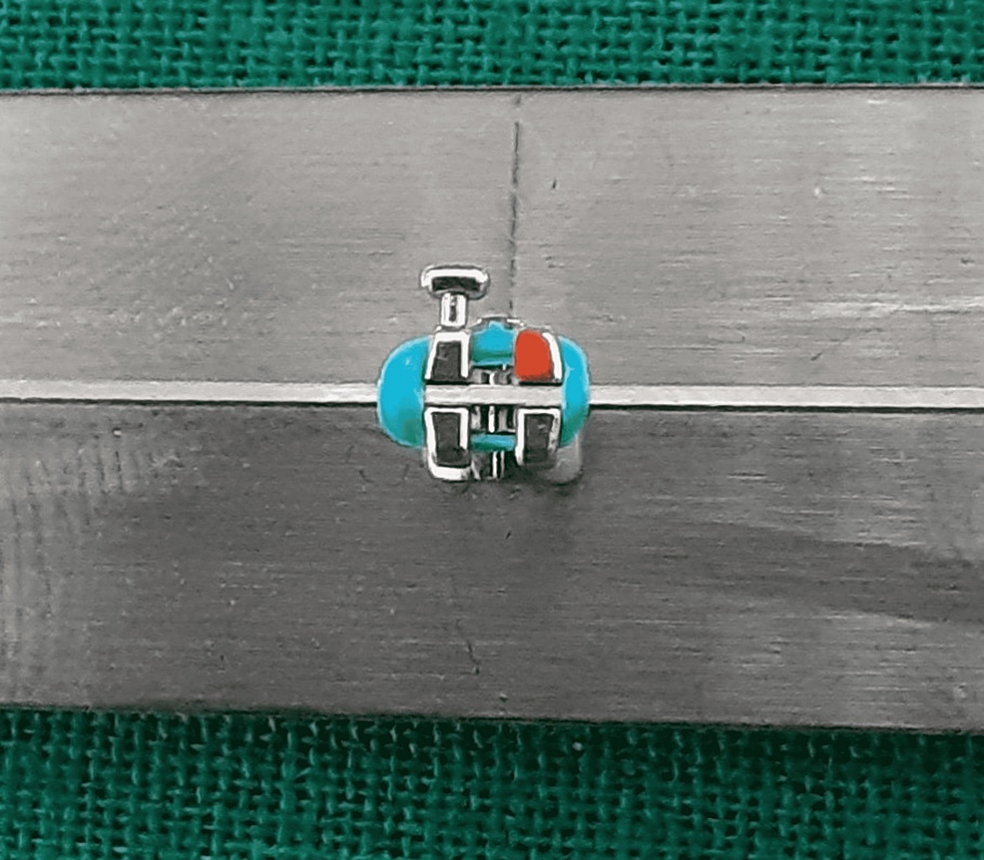
Stainless steel bracket and stainless steel wire combination.

**Figure 8:**
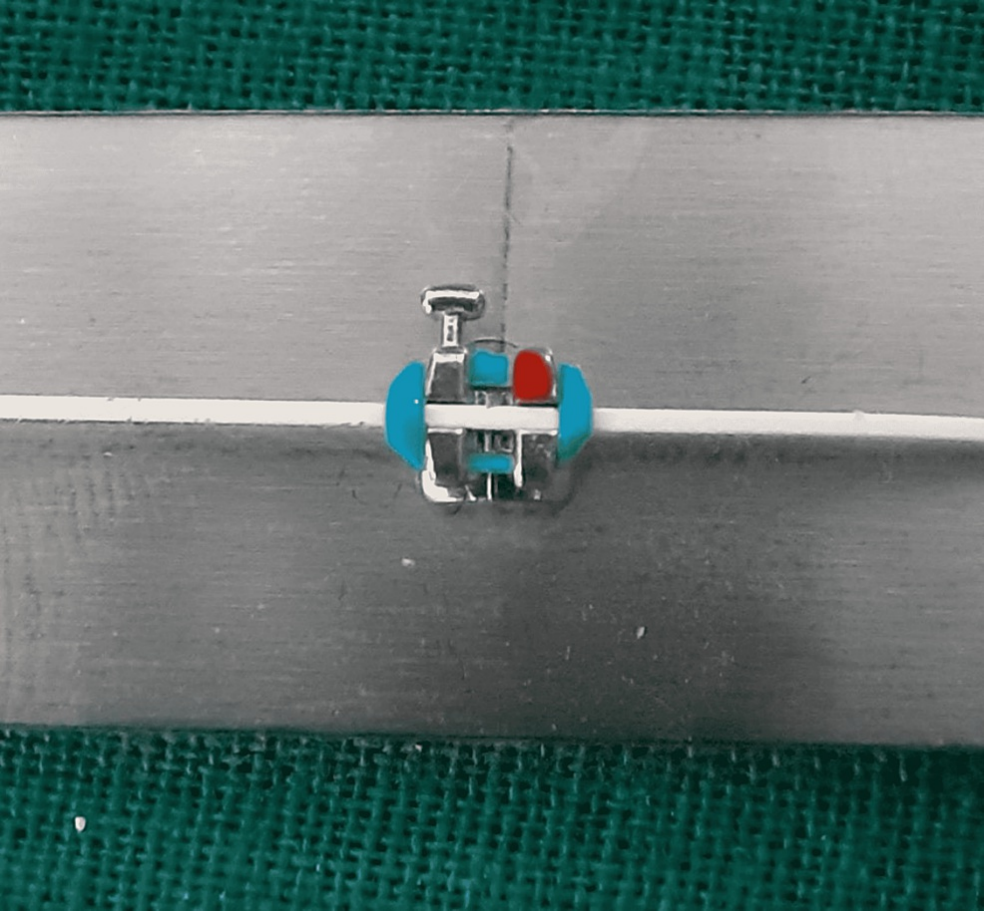
Stainless steel bracket and Teflon-coated stainless steel wire combination.

Friction was measured with a universal testing machine. To measure roughness, a photomicrographic examination of the bracket slots and wires was performed using a scanning electron microscope (SEM). A total of 48 bracket-wire samples were studied. Each bracket was tested only once, and each wire specimen was drawn through one bracket only to eliminate the influence of wear. Different bracket-wire combinations were divided into different groups (Table 3; Figures 1-8).

**Table 3:** The different bracket-wire combinations used in this study.

Groups	Bracket and wire combination
Group 1A	Monocrystalline ceramic bracket with stainless steel wire
Group 1B	Monocrystalline ceramic bracket with Teflon-coated stainless steel wire
Group 2A	Polycrystalline ceramic bracket with stainless steel wire
Group 2B	Polycrystalline ceramic bracket with Teflon-coated stainless steel wire
Group 3A	Polycrystalline ceramic bracket with a stainless steel slot and stainless steel wire
Group 3B	Polycrystalline ceramic bracket with a stainless steel slot and Teflon-coated stainless steel wire
Group 4A	Stainless steel bracket with stainless steel wire
Group 4B	Stainless steel bracket with Teflon-coated stainless steel wire

A stainless steel bar measuring 100 × 15 × 2 mm was used for mounting the bracket-archwire assembly. A line measuring 1 mm in depth was inscribed in the midline, parallel to the long axis, to act as a guide for reproducible bracket positioning. This was done to help align the pull of the wire through the bracket slot so that friction is not induced by adverse tipping or torsion moments. The different types of ceramic brackets and stainless steel brackets were bonded onto the stainless steel bar with cyanoacrylate gum using a bracket placer and 0.021 × 0.025-inch stainless steel jig.

**Figure 9:**
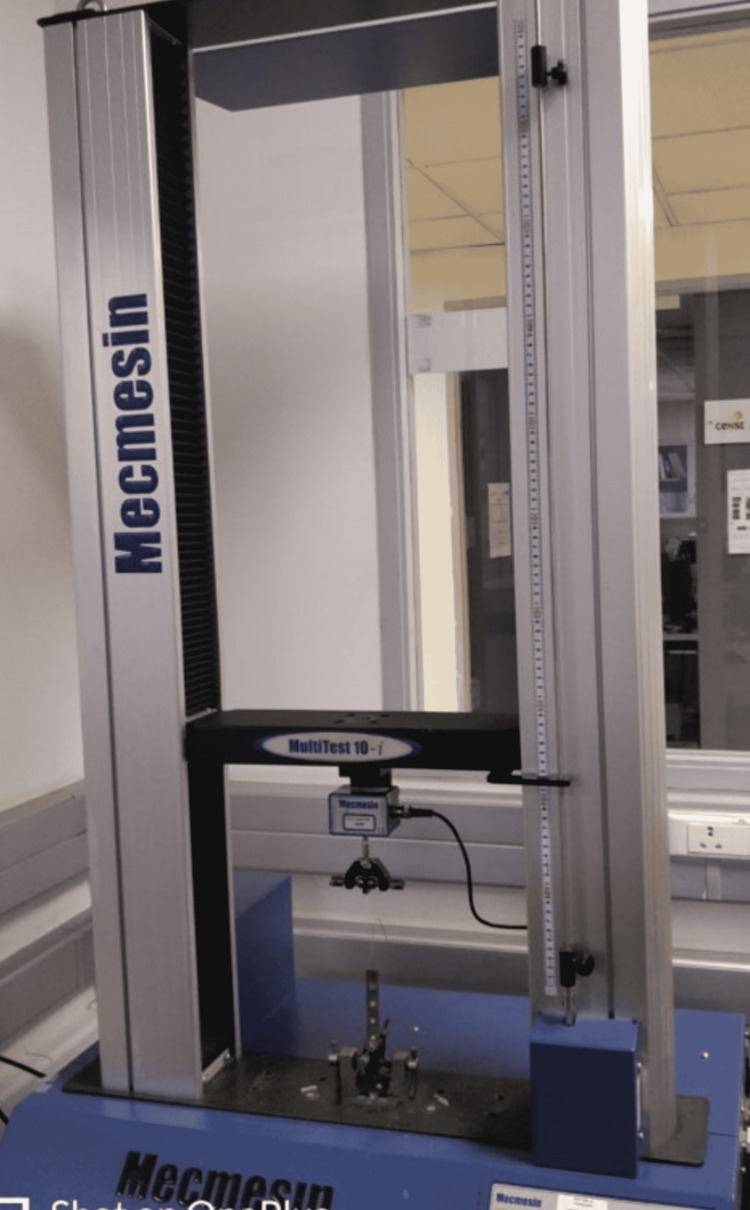
Mecmesin universal testing machine with the archwire and metal bar held by clamps.

Stainless steel (Ortho technology, Carlsbad, CA, USA) and Teflon-coated stainless steel archwire (Ortho technology, Carlsbad, CA, USA) segments were then ligated to the brackets with the help of elastomeric modules (Ortho technology, Carlsbad, CA, USA). One end of the stainless steel bar was attached to the universal testing machine using clamps and the other end was suspended free (Figure 9). The archwire was pulled through the slot with a crosshead speed of 10 mm/minute. Each wire segment was passed through the bracket once. The readings of the tests were displayed and recorded on the computer system attached to the testing apparatus. The readings obtained by this method were used to determine the frictional resistance offered by that combination of wire and bracket. This procedure was repeated for all the bracket and wire combinations.

**Figure 10:**
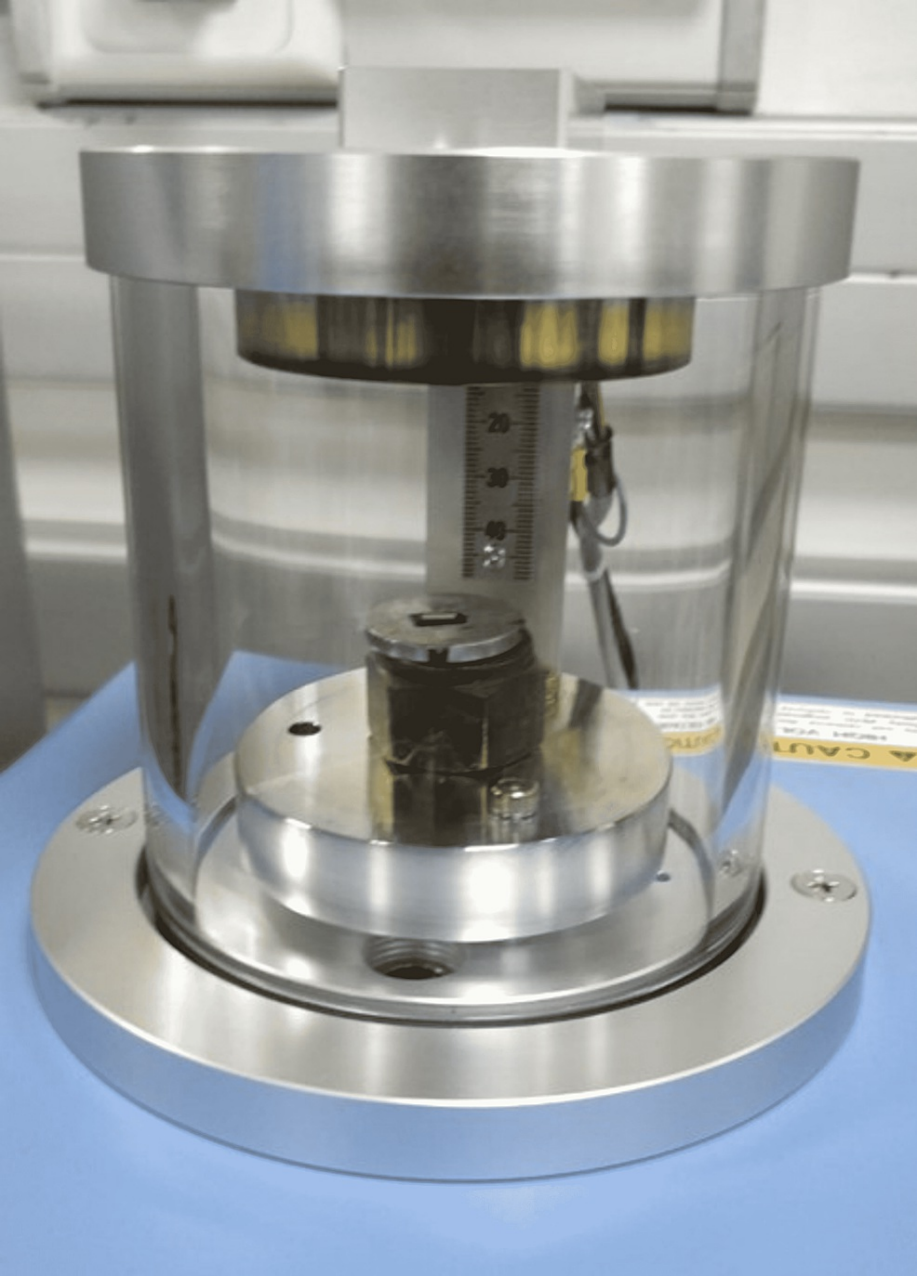
Gold sputtering done for Teflon-coated wire to increase its conductivity.

**Figure 11:**
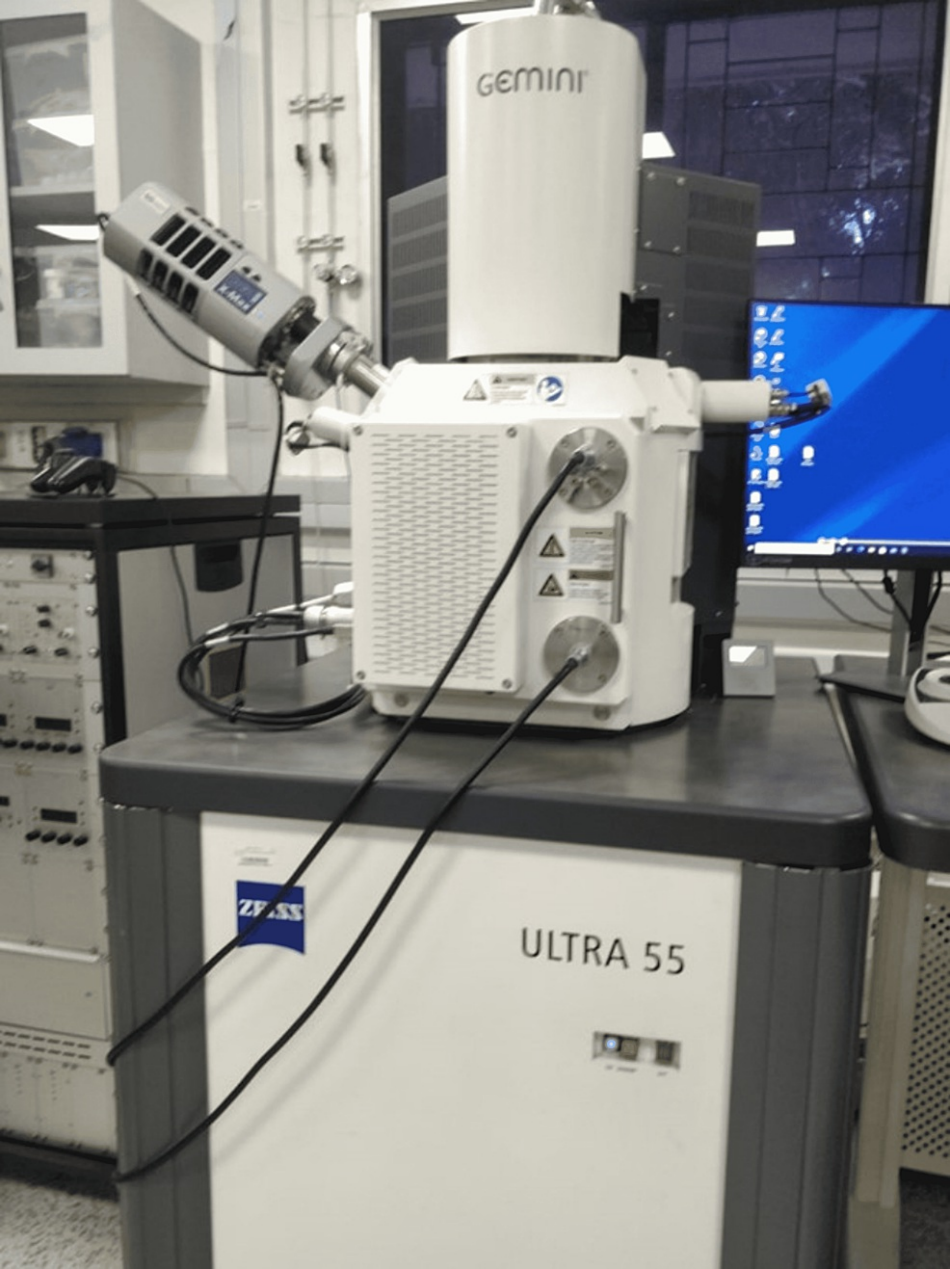
Scanning electron microscope.

The surface roughness of the stainless steel and Teflon-coated stainless steel wires were examined before and after testing for friction using an SEM. The stainless steel and Teflon-coated stainless steel wires were cut to 10 mm in length. As Teflon-coated stainless steel wire is nonconductive, gold sputtering had to be done before examining surface characteristics using SEM (Figure 10). Both wires were placed onto the studs of the SEM machine, thereby allowing examination of surface characteristics of the wire such as surface roughness. The surface roughness was examined at magnifications of 250×, 500×, 1,000×, and 5,000× (Figure 11). After the friction test, the portion of the wire that passed through the bracket slot was cut to 10 mm in length, and the procedure was repeated.

Statistical analysis

The data were tabulated in Microsoft Excel 2010 and statistical analysis was performed. Descriptive statistics were presented as mean, standard deviation (SD), minimum, and maximum values. The values were tested for normality using the Shapiro-Wilk test. Because the data were not normally distributed, The Kruskal-Wallis test with post hoc test was performed for inferential statistics. P-values less than 0.05 were considered statistically significant.

## Results

Friction measurement using the Kruskal-Wallis test showed a significant bracket effect (p < 0.001). Post hoc pairwise comparisons showed that the polycrystalline ceramic bracket had the highest frictional force, which was statistically significant, followed in decreasing order by monocrystalline ceramic bracket, ceramic bracket with a metal slot, and stainless steel bracket. The Kruskal-Wallis test showed a significant wire alloy effect (p < 0.001). Post hoc pairwise comparisons showed that the Teflon-coated stainless steel archwire produced frictional resistance comparable to the stainless steel archwire (Table 4).

**Table 4:** Descriptive statistics: frictional resistance (Newtons). SD: standard deviation

Groups	Mean (N)	SD (N)	Minimum (N)	Maximum (N)
Group 1: polycrystalline ceramic with Teflon-coated wire	2.87	2.4712	1.49	7.83
Group 2: polycrystalline ceramic with stainless steel wire	3.13	2.0246	0.66	6.42
Group 3: monocrystalline ceramic with Teflon-coated wire	3.1	1.697	1.49	6.26
Group 4: monocrystalline ceramic with stainless steel wire	2.27	0.9732	1.01	3.48
Group 5: ceramic with a metal slot with Teflon-coated wire	1.03	0.1879	0.88	1.3
Group 6: ceramic with a metal slot with stainless steel	1.61	0.9042	0.61	3.32
Group 7: stainless steel bracket with Teflon-coated wire	0.92	0.4694	0.37	1.65
Group 8: stainless steel bracket with stainless wire	0.81	0.3541	0.35	1.3

**Figure 12:**
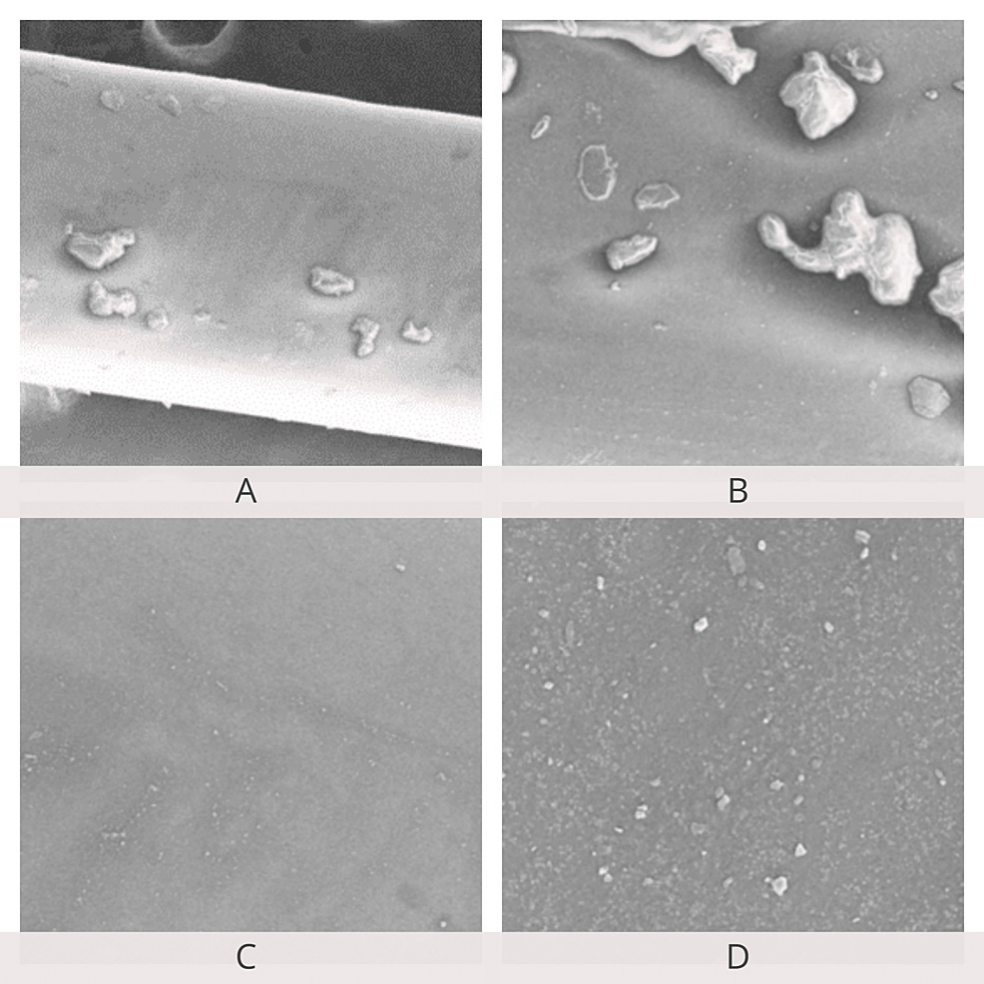
Scanning electron microscopy of stainless steel wires before the friction test. A: 250×; B: 500×; C: 1,000×; D: 5,000× magnification.

**Figure 13:**
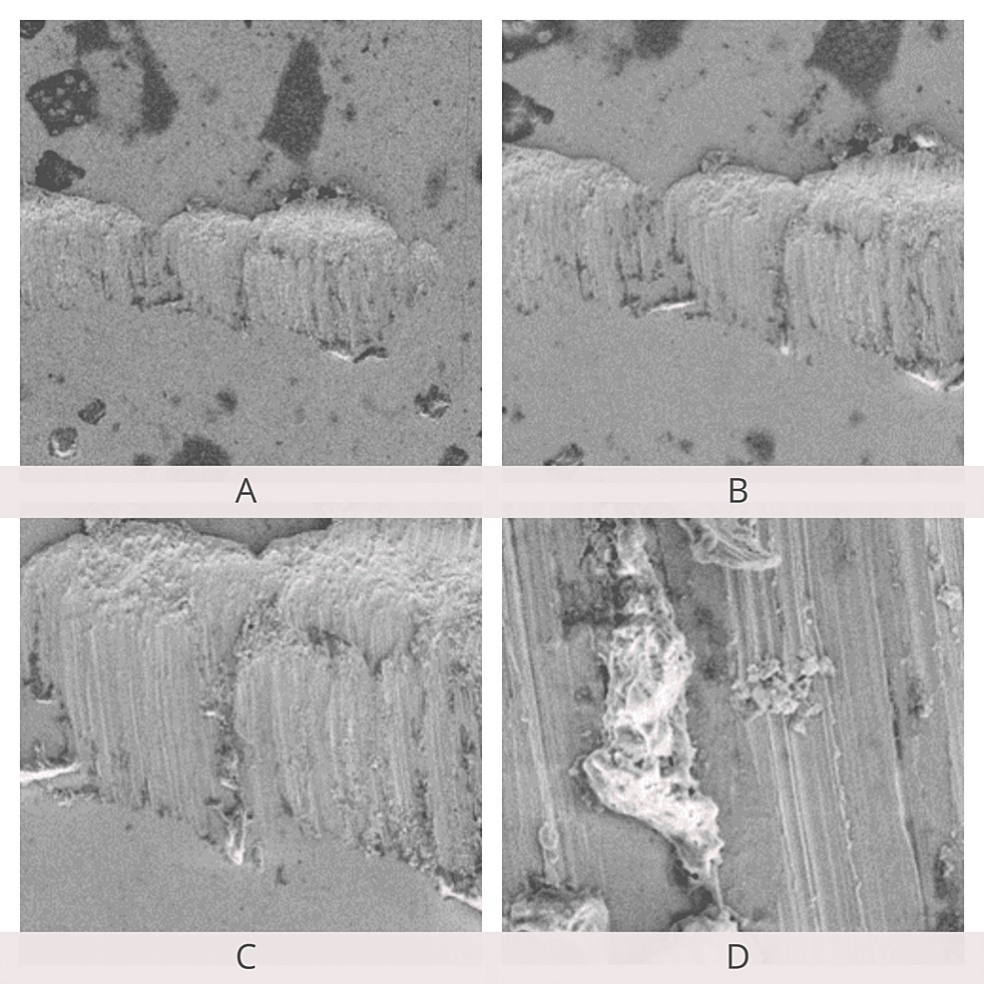
Scanning electron microscopy of stainless steel wires after the friction test. A: 250×; B: 500×; C: 1,000×; D: 5,000× magnification.

**Figure 14:**
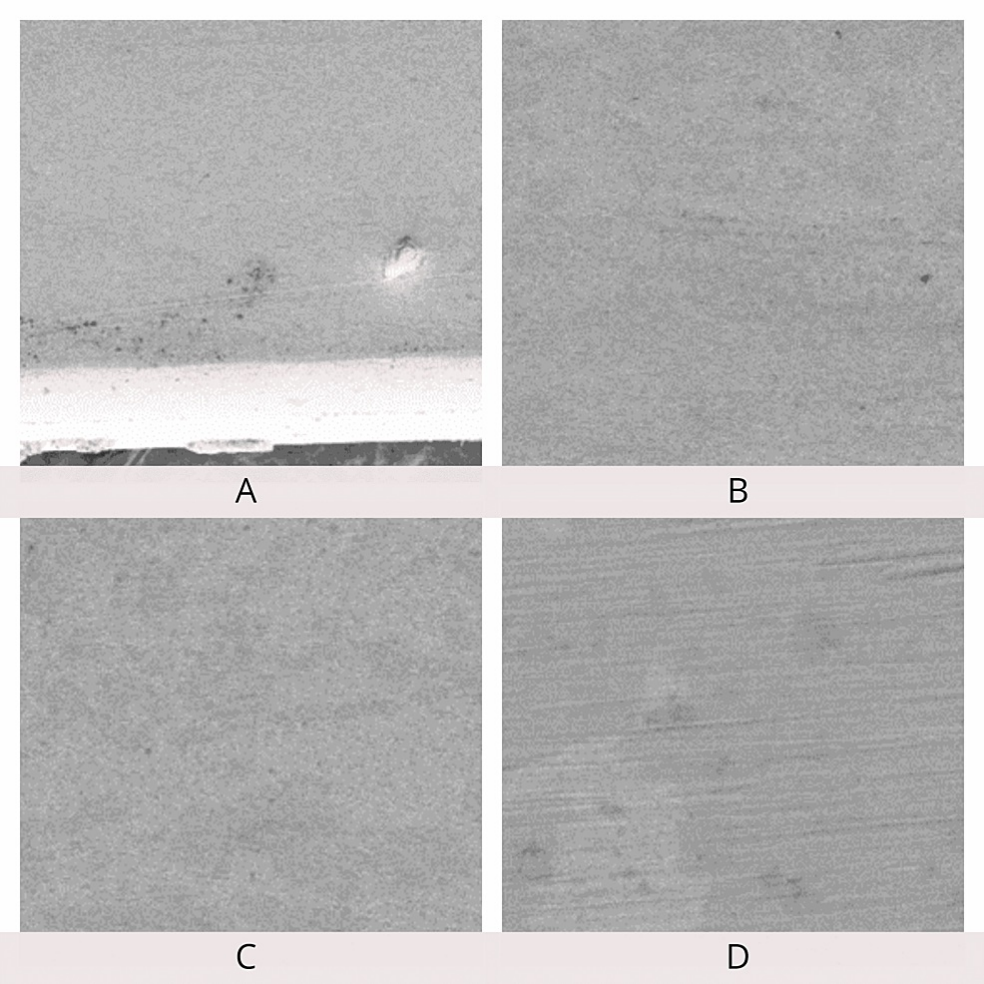
Scanning electron microscopy of Teflon-coated stainless steel wires before the friction test. A: 250×; B: 500×; C: 1,000×; D: 5,000× magnification.

**Figure 15:**
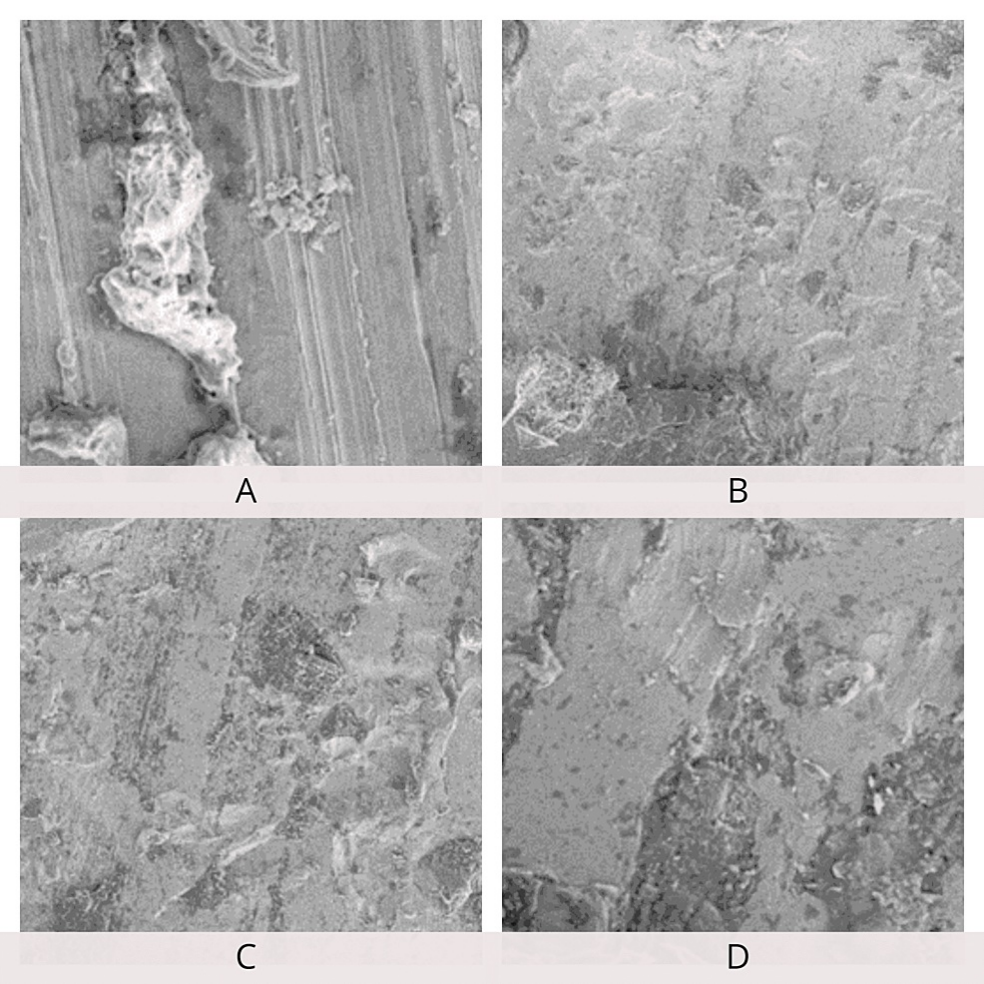
Scanning electron microscopy of Teflon-coated stainless steel wires after the friction test. A: 250×; B: 500×; C: 1,000×; D: 5,000× magnification.

SEM examination of the archwire surfaces at 250×, 500×, 1,000×, and 5,000× magnifications showed a smoother surface of the Teflon-coated stainless steel archwire when compared to conventional stainless steel archwire before the friction test. Archwire roughness and frictional resistance showed a positive correlation. SEM examination after the friction test revealed an increase in the surface roughness of Teflon-coated stainless steel archwire compared to stainless steel archwire (Figures 12-15).

## Discussion

Most kinds of orthodontic treatment often have some form of sliding mechanics to move the teeth. Sliding mechanics can be used to align irregular teeth or close extraction spaces. Friction is inadvertently present between the bracket-wire assembly, thereby reducing the efficiency of orthodontic treatment. Therefore, frictional forces must be overcome to produce tooth movement during alignment and space closure. Frictional forces arise between two surfaces in contact when there is relative motion between the two, or the potential for it, and when the contacting surfaces are not perfectly smooth. The magnitude and variability of the frictional forces determine the efficiency of treatment and the reproducibility of results when similar activation forces are applied. Efficient orthodontic tooth movement requires the application of light continuous forces. To apply light forces, the selection of materials is important. Orthodontists can choose from an extensive inventory of materials such as metal, ceramic, polymer, and composite. Each archwire-bracket combination has a unique set of physical properties that may make it perform differently from others [8].

The current study was undertaken to evaluate the static friction of stainless steel and Teflon-coated stainless steel wires measuring 0.019 × 0.025 inches combined with stainless steel, polycrystalline ceramic, polycrystalline ceramic bracket with a metal slot, and monocrystalline ceramic bracket (0.022 × 0.028 inches). A sample size of 48 was calculated using statistical analysis, which was divided into eight groups based on the bracket-wire combination. Each test specimen was tested for frictional resistance in the universal testing machine, and the same test specimen was evaluated under an SEM to check for surface roughness before and after the friction test.

The results of this study show that there is a significant difference in frictional resistance when different combinations of brackets and archwires are used. Thus, the null hypothesis that frictional resistance is not affected by the different bracket and archwire materials was rejected. The mean frictional resistance ranges from 0.81 to 3.13 N. The highest frictional resistance was displayed by polycrystalline bracket and stainless steel wire combination (3.13 N), and the lowest frictional resistance was displayed by stainless steel bracket and stainless steel wire combination (0.81 N). For ceramic brackets used in this study, both polycrystalline and monocrystalline brackets in combination with stainless steel archwire showed high frictional resistance compared to conventional stainless steel bracket and stainless steel archwire combination. This is in accordance with the study conducted by Fidalgo et al. on friction between the different wire and bracket combinations in artificial saliva. The authors reported that there was a significant difference in friction between ceramic bracket-stainless steel archwire combination and stainless steel bracket-stainless steel archwire combination [9]. Pratten et al. reported that ceramic brackets provide significantly greater frictional resistance than stainless steel brackets when used in combination with either stainless steel or nitinol archwires. The ceramic brackets were originally introduced due to increased esthetic demand from orthodontic patients. However, due to high frictional resistance, their use in regular practice was questionable. This led to several modifications in their design such as the incorporation of a metal slot [10].

In this study, two groups contained polycrystalline ceramic brackets with a metal slot in combination with stainless steel wire and Teflon-coated stainless steel wire. Both the groups have shown superior results compared to polycrystalline and monocrystalline ceramic bracket groups by displaying reduced frictional resistance. These results are supported by the study conducted by Chaa et al. on the friction of conventional and silica-insert ceramic brackets in various bracket-wire combinations. They concluded that the polycrystalline alumina bracket with a metal slot showed significantly lower friction than ceramic brackets [11].

Two types of archwires, stainless steel, and Teflon-coated stainless steel wire were used in this study in combination with different types of brackets. The results of this study show that the wire-bracket combinations with Teflon-coated stainless steel archwire had significantly less frictional resistance when compared to stainless steel wire. These results correspond with the study conducted by Farronato et al. They concluded that, for all bracket-archwire combinations, Teflon-coated archwires resulted in lower friction than the corresponding uncoated archwires, and the results showed that Teflon coating has the potential to reduce resistance to the sliding of orthodontic archwires [12].

Another important characteristic of orthodontic wires is their surface roughness. It affects various factors such as friction, the amount of microbial plaque accumulation, and corrosion of wires, which, in turn, plays a role in orthodontic treatment [13]. Teflon coating and ion implantation are the most common techniques used to improve the surface characteristics of wires. From an orthodontic point of view, Teflon is an anti-adherent and esthetic material with excellent chemical inertia as well as good mechanical stability. These processes can decrease surface roughness. Hence, coating wires with soft materials makes their surface smoother and facilitates easier sliding movements between the wire and the bracket. This study aimed to determine and compare the surface roughness of coated and uncoated orthodontic wires.

The results of this study showed that Teflon-coated stainless steel wires show lesser surface roughness compared to stainless steel wires, which is in accordance with a study conducted by Mousavi et al. to evaluate the effect of esthetic coating on the surface roughness of orthodontic archwires. They compared the surface roughness of four coated esthetic wires with that of a conventional orthodontic wire and concluded that coated wires have significantly less frictional resistance compared to conventional uncoated wires [13]. Rudge et al. also reported that coated orthodontic wires exhibited less surface roughness compared to uncoated wires [12]. Similar results have also been reported by other researchers reporting smoother surfaces in coated archwires of different brands [13].

In this study, the post-frictional test SEM analysis of Teflon-coated archwire showed increased surface irregularities compared to that of stainless steel archwire. Therefore, the Teflon-coated stainless steel wire likely generates higher frictional resistance than stainless steel wire when used for a longer duration. This increase in frictional resistance can be attributed to the increase in surface roughness due to distortion of the coating during the sliding of the archwire through the bracket [13].

Therefore, considering all the above-mentioned findings, the stainless steel bracket-stainless steel wire combination generated the least frictional resistance, and the polycrystalline ceramic bracket-stainless steel wire generated the highest frictional resistance. The surface roughness of Teflon-coated wires was found to be smoother than the conventional uncoated stainless steel wire.

The limitations of this study are that the influence of saliva, temperature, humidity, changes in the ligation force, inter-bracket distance, and movements occurring during mastication were not considered. All these factors cause variations in the frictional values. Nevertheless, the results obtained in this in vitro study can be carefully generalized to clinical conditions as a useful guide for the selection of bracket-archwire combinations to obtain good sliding mechanics in orthodontic applications.

## Conclusions

The stainless steel bracket and stainless steel archwire combination generated the least frictional resistance. The stainless steel bracket with Teflon-coated stainless steel wire combination produced the second lowest frictional resistance and was comparable with that of the stainless steel bracket and stainless steel wire combination. Therefore, Teflon-coated stainless steel wires can be used as an alternative to conventional stainless steel wire during retraction using stainless steel brackets as they are more esthetic. All types of ceramic brackets produced higher frictional resistance compared to stainless steel brackets. Ceramic brackets with a metal slot generated lower friction with both stainless steel and Teflon-coated wire, which was comparable to that produced by the stainless steel bracket-stainless steel archwire combination.

The surface characteristics of Teflon-coated stainless steel wire were smoother compared to stainless steel archwire before the friction test. The surface of Teflon-coated stainless steel wire was more irregular compared to conventional stainless steel when observed using SEM after the friction test. Therefore, friction may increase following the use of Teflon-coated stainless steel wire for long durations.
